# Supplementary material for: Coralgal reef morphology records punctuated sea-level rise during the last deglaciation
Source: Nat Commun. 2017 Oct 19;8:1046. doi: 10.1038/s41467-017-00966-x (PMC5648809; doi:10.1038/s41467-017-00966-x)
Supplement: Supplementary file 1 — Supplementary Information [file 41467_2017_966_MOESM1_ESM.pdf]

Supplementary Figure 1

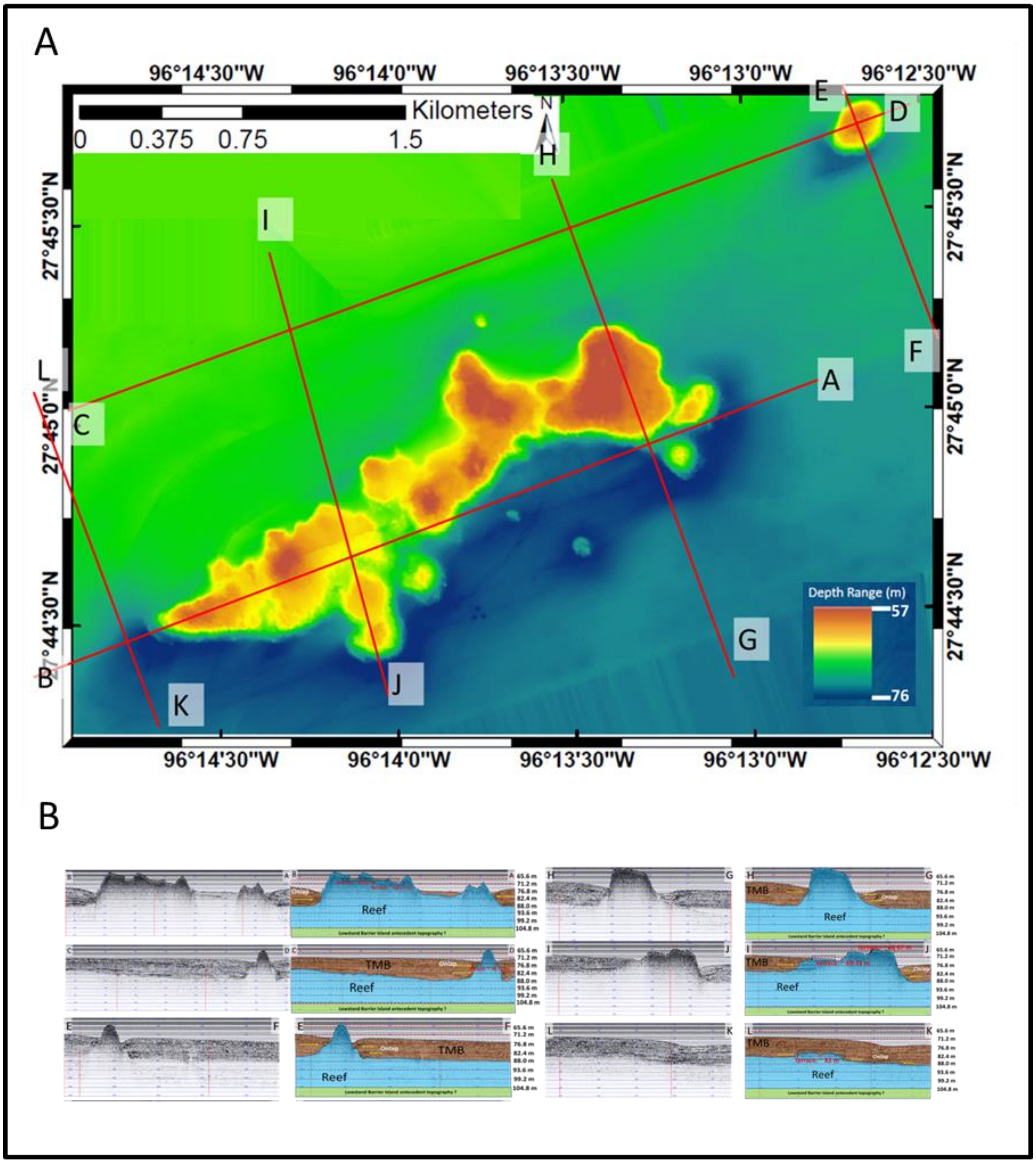

Baker Bank- A) Bathymetric Map: About 19 m of relief of the bank is exposed. Seismic lines A-B, C-D, E-F, G-H I-J and K-L are located. B) 3.5 kHz profiles: uninterpreted and interpreted seismic lines displaying locations of exposed and buried terraces. The bank is buried by TMB which is clearly observed through the seismic lines.

Supplementary Figure 2

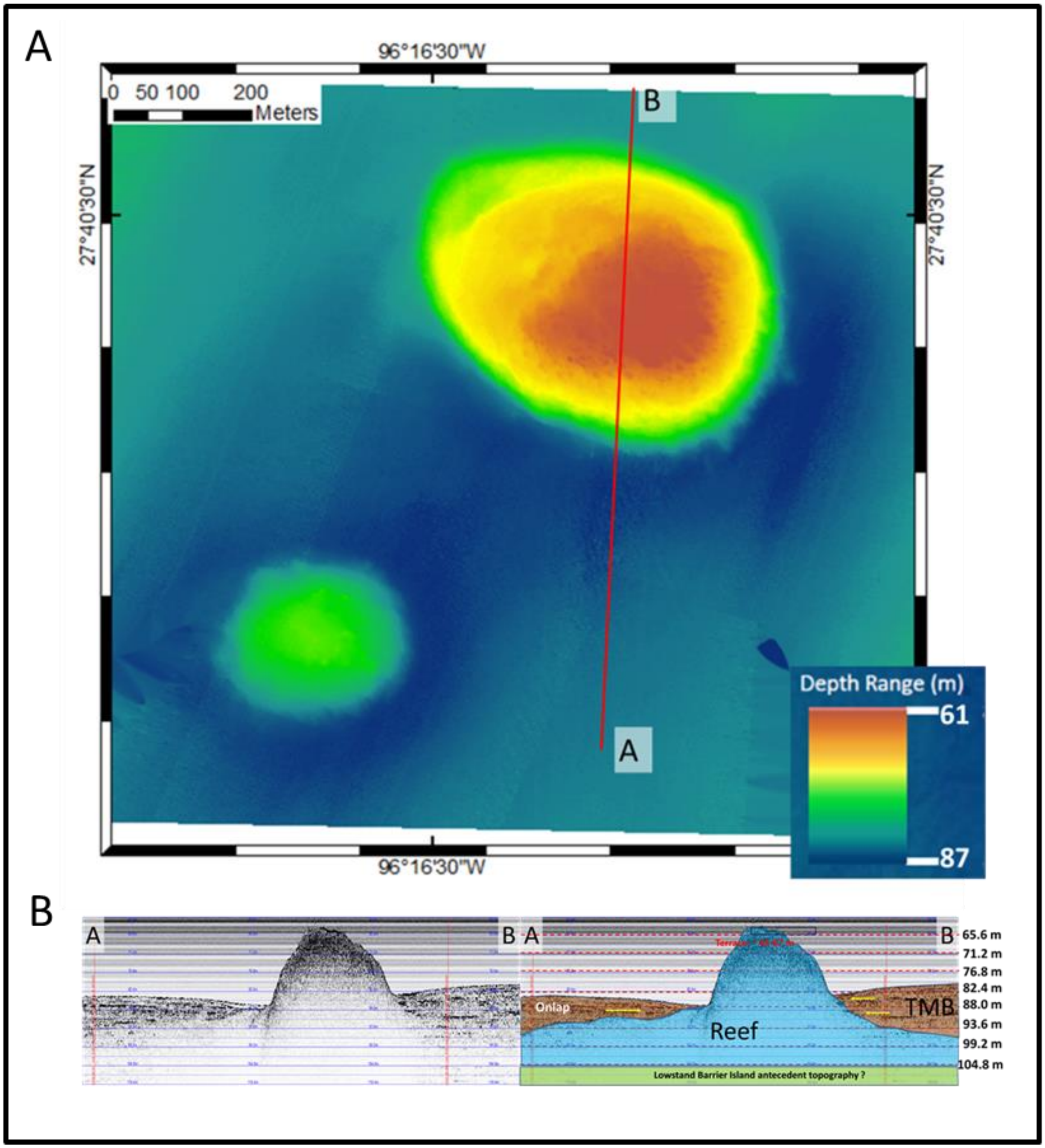

South Baker Bank - Bathymetric Map: About 16 m of bank relief is exposed. Seismic line A-B is located. B) 3.5 kHz profiles: uninterpreted and interpreted seismic lines displaying locations of exposed terrace.

Supplementary Figure 3

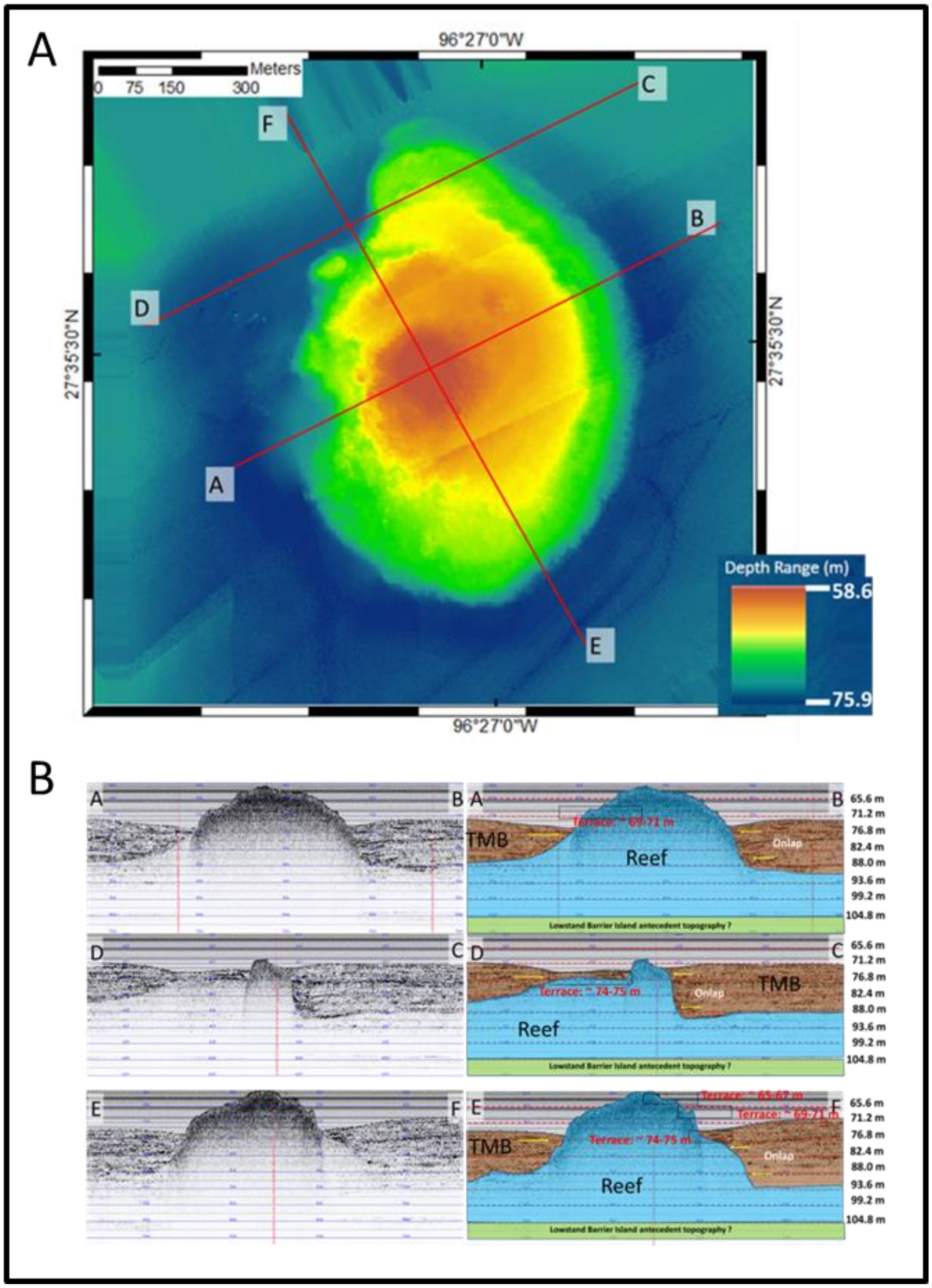

Aransas Bank - A) Bathymetric Map: About 16 m of the bank relief is exposed. Seismic line A-B, C-D, and E-F are located. B) 3.5 kHz profiles: uninterpreted and interpreted seismic lines displaying locations of exposed and buried terraces.

Supplementary Figure 4

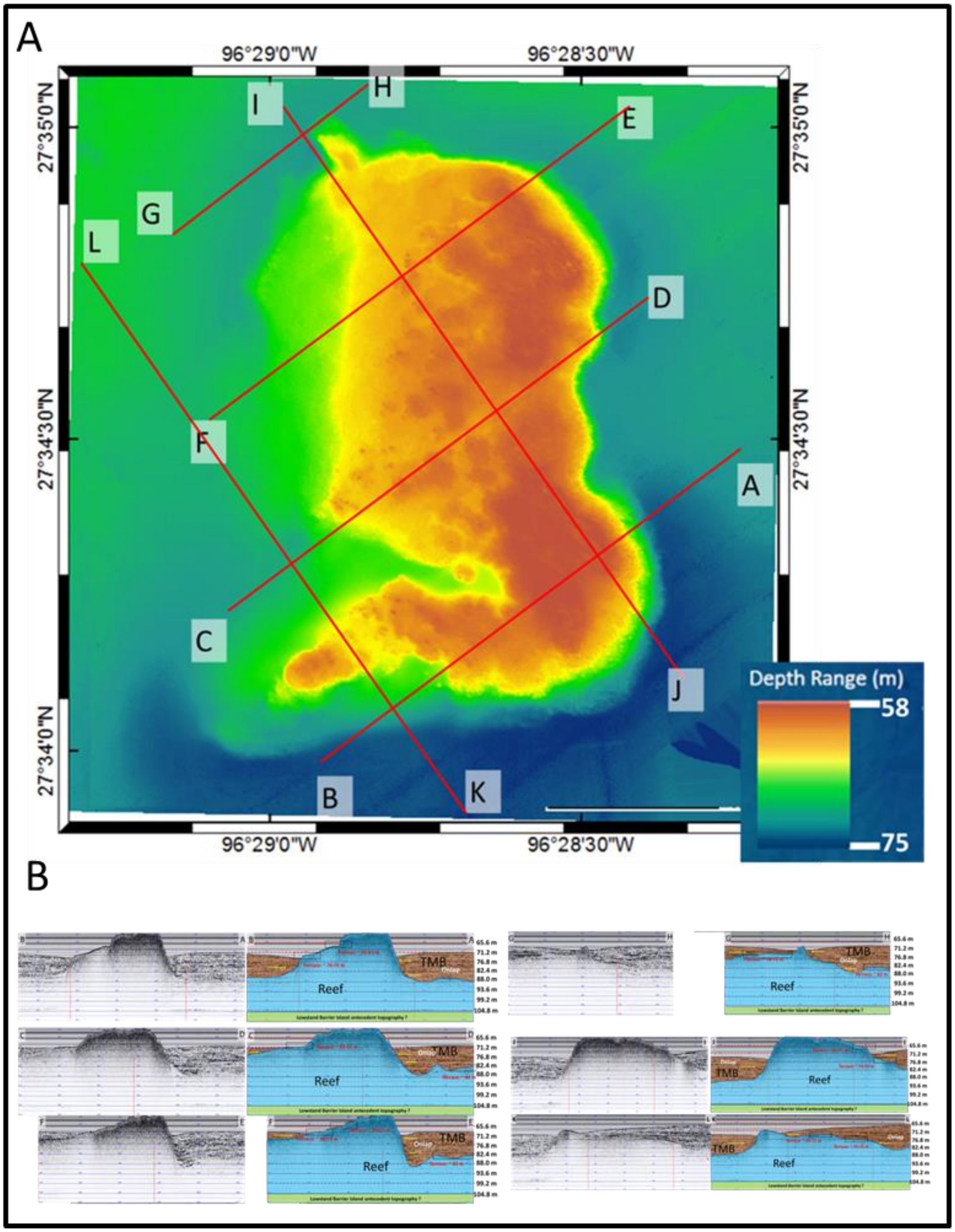

North Hospital Bank- A) Bathymetric Map: About 17 m of bank relief is exposed. Seismic lines A-B, C-D, E-F, G-H, I-J, and K-L are located. B) 3.5 kHz profiles: uninterpreted and interpreted seismic lines displaying locations of exposed and buried terraces.

Supplementary Figure 5

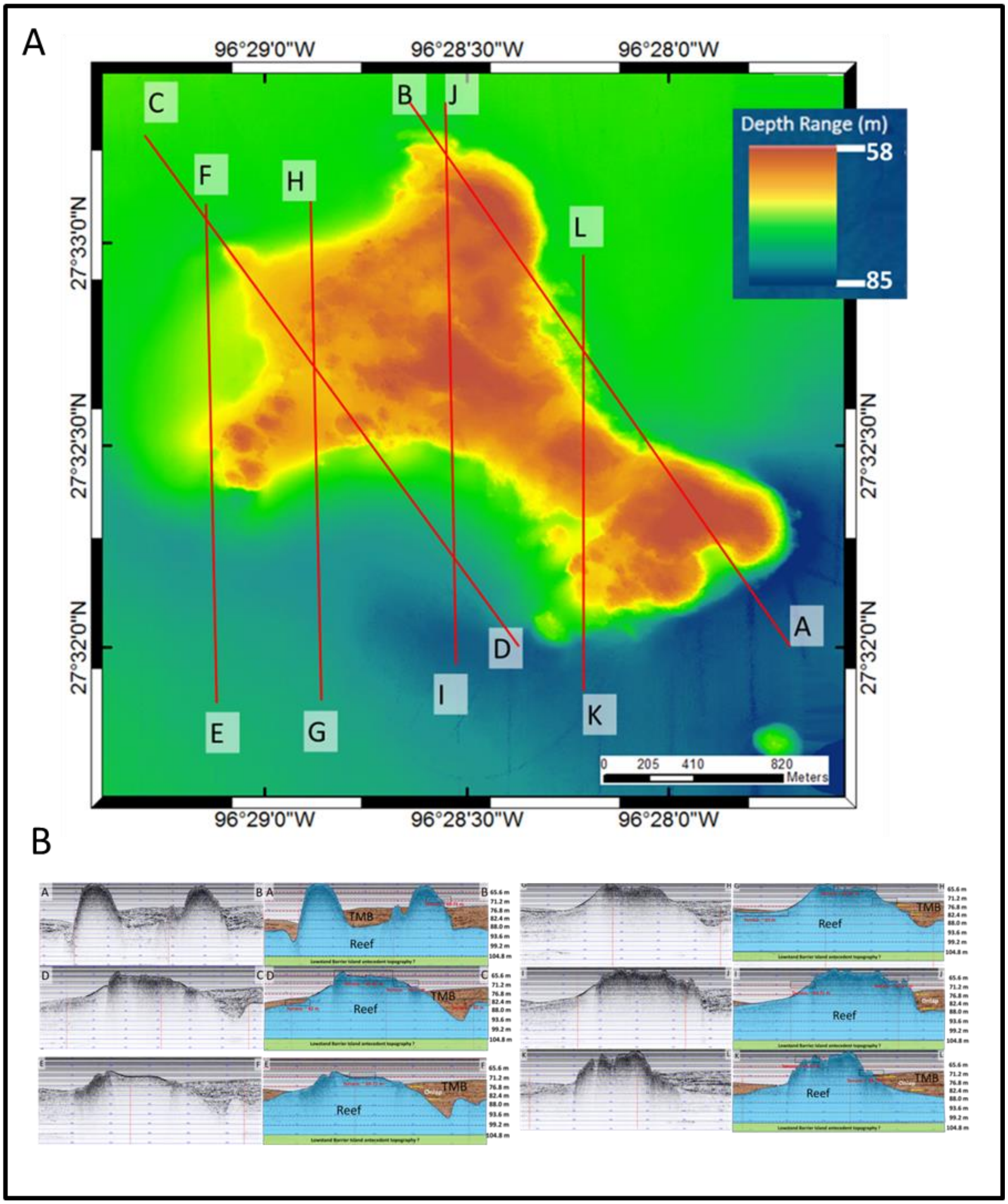

Hospital Bank -A) Bathymetric Map: About 27 m of bank relief is exposed. Seismic lines A-B, C-D, E-F, G-H, I-J, and K-L are located. B) 3.5 kHz profiles: uninterpreted and interpreted seismic lines displaying locations of exposed and buried terraces.

Supplementary Figure 6

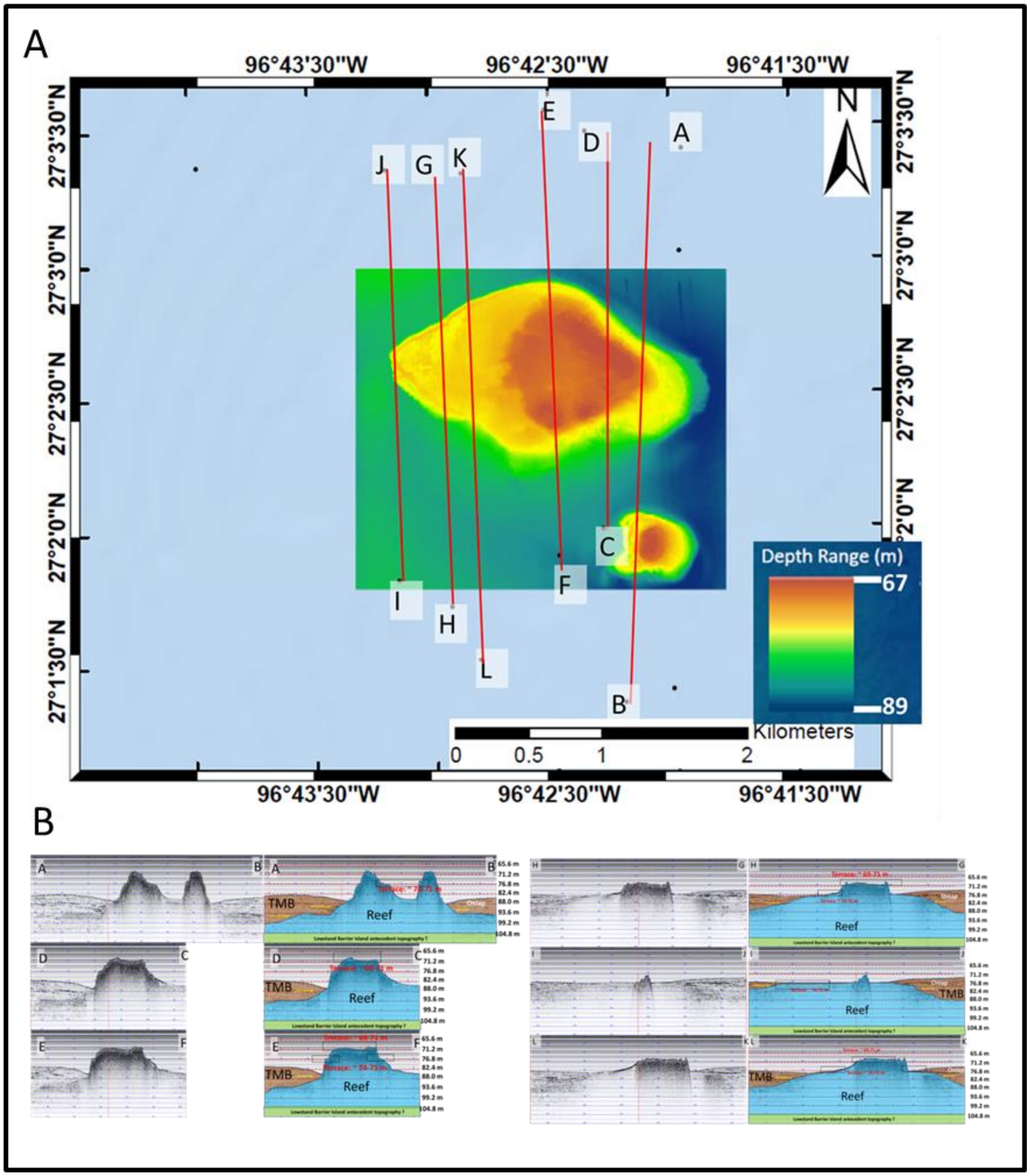

Dream Bank - A) Bathymetric Map: About 22 m of bank relief is exposed. Seismic lines A-B, C-D, E-F, G-H, I-J, and K-L are located. B) 3.5 kHz profiles: uninterpreted and interpreted seismic lines displaying locations of exposed and buried terraces.

Supplementary Figure 7

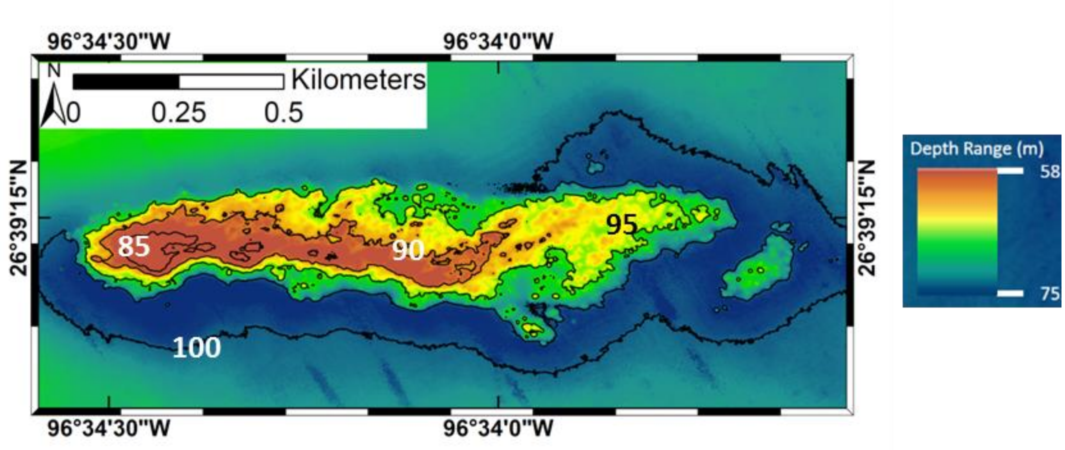

Harte Bank Bathymetric Map: About 21 m of bank relief is exposed.
